# Cetuximab and anemia prevention in head and neck cancer patients undergoing radiotherapy

**DOI:** 10.1186/s12885-022-09708-9

**Published:** 2022-06-07

**Authors:** Lucas Maahs, Ahmed I. Ghanem, Radhika Gutta, Amy Tang, Swarn Arya, Zaid Al Saheli, Haythem Ali, Steven Chang, Samantha Tam, Vivian Wu, Farzan Siddiqui, Jawad Sheqwara

**Affiliations:** 1 Department of Internal Medicine, Henry Ford Hospital, Detroit, MI 48202 USA; 2 Department of Radiation Oncology, Henry Ford Cancer Institute, Detroit, MI 48202 USA; 3 Department of Clinical Oncology, Faculty of Medicine, Alexandria University, Alexandria, Egypt; 4 Department of Public Health Sciences, Henry Ford Health System, Detroit, MI USA; 5 Department of Hematology and Medical Oncology, Henry Ford Cancer Institute, Detroit, MI 48202 USA; 6 Department of Otolaryngology, Henry Ford Cancer Institute, Detroit, MI 48202 USA

**Keywords:** Head and neck cancer, Squamous cell carcinoma, Concomitant cetuximab, Radiation therapy, Radiotherapy induced anemia, Anemia, Hemoglobin, Survival

## Abstract

**Background:**

Epidermal growth factor receptor (EGFR) activation is associated with increased production of interleukin 6 (IL6), which is intensified by radiotherapy (RT) induced inflammatory response. Elevated IL6 levels intensifies RT-induced anemia by upregulating hepcidin causing functional iron deficiency. Cetuximab, an EGFR inhibitor, has been associated with lower rates of anemia for locally advanced head and neck squamous cell carcinoma (HNSCC). We hypothesized that concomitant cetuximab could prevent RT-induced anemia.

**Methods:**

We queried our institutional head and neck cancers database for non-metastatic HNSCC cases that received RT with concomitant cetuximab or RT-only between 2006 and 2018. Cetuximab was administered for some high-risk cases medically unfit for platinum agents per multidisciplinary team evaluation. We only included patients who had at least one complete blood count in the 4 months preceding and after RT. We compared the prevalence of anemia (defined as hemoglobin (Hb) below 12 g/dL in females and 13 g/dL in males) and mean Hb levels at baseline and after RT. Improvement of anemia/Hb (resolution of baseline anemia and/or an increase of baseline Hb ≥1 g/dL after RT), and overall survival (OS) in relation to anemia/Hb dynamics were also compared.

**Results:**

A total of 171 patients were identified equally distributed between cetuximab-plus-RT and RT-only groups. The cetuximab-plus-RT group had more locally-advanced stage, oropharyngeal and high grade tumors (*p < 0.001* for all). Baseline anemia/Hb were similar, however anemia after RT conclusion was higher in the cetuximab-plus-RT vs RT-only (63.5% vs. 44.2%; *p = 0.017*), with a mean Hb of 11.98 g/dL vs. 12.9 g/dL; *p = 0.003,* for both respectively. This contributed to significantly worse anemia/Hb improvement for cetuximab-plus-RT (18.8% vs. 37.2%; *p = 0.007*). This effect was maintained after adjusting for other factors in multivariate analysis. The prevalence of iron, vitamin-B12 and folate deficiencies; and chronic kidney disease, was non-different. Baseline anemia was associated with worse OS (*p = 0.0052*) for the whole study cohort. Nevertheless, improvement of anemia/Hb was only marginally associated with better OS (*p =* 0.068).

**Conclusions:**

In contrast to previous studies, cetuximab was not associated with lower rates of anemia after RT for nonmetastatic HNSCC patients compared to RT-alone. Dedicated prospective studies are needed to elucidate the effect of cetuximab on RT-induced anemia.

**Supplementary Information:**

The online version contains supplementary material available at 10.1186/s12885-022-09708-9.

## Background

Anemia is a major complication of cancer, as well as many of its treatment options, and can be a cause of significant morbidity in oncologic patients [1–5]. The prevalence rates of anemia vary depending on the type of cancer, cancer stage and definition of anemia, but rates of up to 90% have been reported [1]. Radiation therapy (RT) can induce anemia or worsen a pre-existing anemia, and this effect is accentuated if concomitant systemic therapy is administered. A study by Harrison at al. reported that 48% of patients presenting for RT had anemia and 57% were anemic at the end of therapy [2]. In head and neck cancer, the prevalence of anemia defined as hemoglobin (Hb) < 12.0 g/dL has been reported as 16% prior to treatment and 32% within 3–5 weeks after the first RT dose, resulting in a mean Hb decrease of 1.8 g/dL [3, 4]. Additionally, anemia may worsen the response of some cancers to RT. The solid tumor microenvironment is hypoxic compared to non-diseased tissue, which is more pronounced in head and neck cancers, and tumor hypoxia has been previously associated with dismal outcomes and decreased sensitivity to RT. Anemia is thought to worsen intramural hypoxia and its presence before or during RT adversely impacts tumor radiosensitivity and is independently associated with poor locoregional disease control and survival [4, 6–8]. Hence, many studies focused on the mitigation of tumor hypoxia using various local and systemic modalities including the correction of baseline Hb concentration before and during the RT course. Treatment of cancer-related anemia relies on identifying the cause (nutritional deficiencies, chronic kidney disease, hemorrhage, hemolysis, inherited, treatment-induced) and managing it accordingly [1]. Other than that, therapeutic options are limited and rely mainly on blood transfusions and, to a smaller degree, erythropoietin stimulating agents, both of which carry significant risks of adverse events and were not proven to enhance oncologic outcomes after anemia correction [9]. The use of erythropoietin stimulating agents is significantly decreasing mainly due to concerns that the therapy may facilitate disease progression, mainly locoregionally [1, 9, 10].

Epidermal growth factor receptor (EGFR), also referred to as human epidermal growth factor-1 (HER-1), is part of the ErbB family (that includes also HER-2, HER-3 and HER-4). The activation of the receptor by natural ligands, mainly EGF and transforming growth factor alpha (TGF-⍺), promotes activation of the intracellular tyrosine kinase that leads to the inhibition of apoptosis, cell proliferation and angiogenesis [11]. In head and neck cancer, EGFR and TGF-⍺ are overexpressed in 80–90% of cases and are associated with lower rates of locoregional control and survival after RT [6]. Another downstream effect of EGFR activation is increased production of interleukin 6 (IL-6), which can be intensified by RT due to its inflammatory response [12, 13]. IL-6 causes upregulation of hepcidin production, a key protein in the regulation of iron metabolism. Hepcidin increases the trapping of iron in the liver, making it unavailable to hematopoietic tissues and leading to a functional iron deficiency, which could explain the worsening anemia rates seen in patients that undergo RT [2–4, 13]*.*

Cetuximab is an EGFR inhibitor that is used in the treatment of head and neck squamous cell carcinoma (HNSCC). It is an important option concomitant with RT both in the definitive setting [14] and postoperatively in high-risk patients [15], especially for patients that cannot tolerate a platinum-based regimen. It is commonly administered with RT-alone, or combined with a non-platinum agent like docetaxel, and can also be given following induction chemotherapy [16]. The addition of cetuximab to RT has been demonstrated to improve locoregional control when compared to RT-alone, but platinum-based regimens remain standard of care for fit patients [14, 17, 18]. A study by Bonner et al. reported a significant reduction in anemia rates in this setting, raising the question of whether cetuximab can be used for prevention or treatment of RT induced anemia [14]. Another study by Ang et al. showed that adding cetuximab to concurrent cisplatin and radiation did not result in a significant change in anemia [18]. In the recurrent and metastatic settings, adding cetuximab to platinum and 5-fluorouracil chemotherapy resulted in lower rates of anemia, although the difference was not statistically significant [19].

The primary aim of this study was to evaluate whether administration of cetuximab with RT is associated with improved rates of anemia for the treatment of non-metastatic HNSCC. We hypothesized that patients who receive cetuximab with RT would have decreased rates of anemia after treatment compared to patients who received RT-alone.

## Methods

### Data source and patient selection

Patients with nonmetastatic HNSCC that received RT definitively or in the adjuvant setting, with or without cetuximab as a primary treatment between 2006 and 2018 were identified from the prospectively maintained database encompassing all head and neck cancer subjects of Henry Ford Cancer Institute (Detroit, MI, USA). Cetuximab was administered for some high-risk cases that were medically unfit for platinum agents per multidisciplinary team evaluation. Possible factors for this decision include poor renal functions, hearing problems, poor performance status, as well as patient preference. We excluded all patients that received concomitant chemotherapy, induction chemotherapy as well as those with nasopharyngeal cancer and those who failed to complete their planned RT course. Patients were only included if they had at least one complete blood count within 4 months before RT course started, in addition to another one up to 4 months after treatment. The study was approved by the Henry Ford Health System Institutional Review Board (IRB number: 13133) and participation consent waiver was granted due to the retrospective nature of the research.

### Study variables

Patients were divided in two groups: RT-alone vs. RT with cetuximab. Data collected included patient demographics (age, gender, race), Charlson comorbidity index (CCI), smoking and alcohol history, primary tumor site, disease stage per AJCC (early (stages I & II) vs. locally-advanced (stages III &IV)), human papilloma virus (HPV) positivity for oropharyngeal tumors only (according to P16 status) and tumor grade of differentiation for non-HPV related tumors whenever available [20, 21]. Radiological response to RT within 6 months of RT conclusion per RECIST criteria 1.1 and survival status at the last follow up were also gathered [22]. Pre- and post-RT Hb levels were reported from complete blood counts for all the study population. We calculated glomerular filtration rate for all cases pre- and post-RT and if poor renal function persisted chronic kidney disease (CKD) was graded as grade (G)3, G4 or G5 using KDIGO guidelines [23]. In addition, basic anemia studies (vitamin B12 levels, folate levels, iron studies including iron and ferritin levels as well as total iron binding capacity) were recorded whenever available before and/or after RT and were compared.

### Outcome assessment

The primary outcome was the prevalence of anemia after RT conclusion. Anemia was defined as Hb level lower than 12 g/dL in women and 13 g/dL in men [24]. Secondary outcomes included mean Hb level changes and improvement of Hb or anemia after treatment. Improvement of anemia/Hb was defined as either resolution of anemia after RT if anemia was present at baseline and/or an increase of Hb level of at least 1.0 g/dL above baseline, regardless of the presence of baseline anemia. Secondary outcomes also included overall survival (OS) in relation to anemia/Hb dynamics across study groups.

### Statistical analysis

Data was presented as mean (standard deviation (SD)) or median (interquartile range (IQR)) for continuous variables, and frequency (percentage) for categorical variables. Continuous variables were compared using either Student’s t-tests or Wilcoxon’s rank-sum tests, depending on the distribution of the data. Categorical variables were analyzed using Fisher’s exact or chi-square tests, as appropriate. Kaplan-Meier curves were plotted to demonstrate overall survival across study groups with log-rank test used for comparison. Multivariate logistic regression models were performed to examine the associations between pre-RT predictors and the presence of anemia, Hb level and improvement of anemia/Hb at the end of RT course. Results were presented with odds ratios (ORs) and 95% CI confidence intervals (CIs). All tests were 2 sided, with a significance level of 0.05. Analyses were performed using R 4.02.2 (R Foundation for statistical Computing, Vienna, Austria).

## Results

### Patient, pathological and treatment characteristics

A total of 171 patients with non-metastatic HNSCC were included in the analysis. Of those, 86 received RT-alone and 85 received cetuximab plus RT. Baseline characteristics of subjects are shown in Table 1. The cetuximab plus RT group had a lower CCI trend (*p* = 0.082) and a higher proportion of oropharyngeal tumors (65.9% vs 30.2%; *p < 0.001*), locally advanced disease (75.3% vs 40.7%, *p < 0.001*), and poorly differentiated tumors (34.4% vs 7.1%; *p < 0.001*). On the other hand, the RT-alone group had more tumors of the oral cavity and larynx (*p < 0.001*) and had a trend towards more middle-aged patients 50–70 years (66.3% vs 51.8%; *p* = 0.092). Radiotherapy details and treatment response data is shown in Table 2. Most patients were treated definitively (*n =* 105, 61.4%), and the remainder received treatment in the adjuvant (postoperative) setting (*n =* 66, 38.6%) with only a non-significant trend towards more adjuvant cases in the RT-alone arm (45.3% vs 31.8%; *p* = 0.095), with lower RT dose received. Overall, the RT-alone group had significantly better radiologic response to RT (*p = 0.009*) with better OS (2-year OS: 69% vs 48%; *p = 0.0058*) when compared to the cetuximab plus RT (Fig. S1). Nevertheless, this survival advantage was lost in patients who received definitive radiotherapy in a subgroup analysis (2-year OS: 43% vs 68%; *p* = 0.12) (Fig. S2).

**Table 1:** Baseline demographic and tumor characteristics for HNSCC patients receiving radiotherapy with or without concomitant cetuximab

		All (*n=*171)	Cetuximab plus RT (*n=*85)	RT alone (*n=*86)	*P* value
Mean age at Diagnosis in years [SD]		65.51 (11.25)	65.71 (12.20)	65.31 (10.29)	0.821
Age group at Diagnosis in years (n (%))	<50	11 (6.4)	8 (9.4)	3 (3.5)	0.092
	50-70	101 (59.1)	44 (51.8)	57 (66.3)	
	>70	59 (34.5)	33 (38.8)	26 (30.2)	
Gender (n (%))	Female	37 (21.6)	18 (21.2)	19 (22.1)	1
	Male	134 (78.4)	67 (78.8)	67 (77.9)	
Race (n (%))	Black	50 (29.2)	23 (27.1)	27 (31.4)	0.822
	White	117 (68.4)	60 (70.6)	57 (66.3)	
	Other	4 (2.3)	2 (2.4)	2 (2.3)	
Median Total Charlson comorbidity index (range)		1 (0-3)	1 (0-2)	2 (1-3)	0.082
Smoking (n (%))	Never	30 (17.5)	15 (17.6)	15 (17.4)	0.918
	Former	87 (50.9)	42 (49.4)	45 (52.3)	
	Active	54 (31.6)	28 (32.9)	26 (30.2)	
Alcohol use (n (%))	Never	57 (33.3)	28 (32.9)	29 (33.7)	0.926
	Occasional	52 (30.4)	25 (29.4)	27 (31.4)	
	Frequent	62 (36.3)	32 (37.6)	30 (34.9)	
Tumor site (n (%))	Oral cavity	29 (17.0)	11 (12.9)	18 (20.9)	*<0.001*
	Oropharynx	82 (48.0)	56 (65.9)	26 (30.2)	
	Hypopharynx	2 (1.2)	1 (1.2)	1 (1.2)	
	Larynx	58 (33.9)	17 (20.0)	41 (47.7)	
Tumor staging (n (%))	Early	72 (42.1)	21 (24.7)	51 (59.3)	*<0.001*
	Locally advanced	99 (57.9)	64 (75.3)	35 (40.7)	
Tumor grade of differentiation (n (%))	Well	10 (7.5)	3 (4.7)	7 (10)	*<0.001*
	Moderate	62 (46.3)	23 (35.9)	39 (55.7)	
	Poor	27 (20.1)	22 (34.4)	5 (7.1)	
HPV status (oropharyngeal cancers) (n (%))	Positive	37 (64.9)	21 (58.3)	16 (76.2)	0.282
	Negative	20 (35.1)	15 (41.7)	5 (23.8)	

*Abbreviations: HNSCC* Head and neck squamous cell carcinoma, *RT* Radiotherapy, *SD* Standard deviation, *n (%)* Number (percentage), *HPV* Human papilloma virus

**Table 2:** Radiotherapy details, response and survival outcomes for HNSCC patients receiving radiotherapy with or without concomitant cetuximab

		All (*n=*171)	Cetuximab plus RT (*n=*85)	RT alone (*n=*86)	*P* value
RT Setting (n (%))	Adjuvant	66 (38.6)	27 (31.8)	39 (45.3)	0.095
	Definitive	105 (61.4)	58 (68.2)	47 (54.7)	
RT dose category (n (%))	70-72 Gy	101 (59.1)	63 (74.1)	38 (44.2)	*<0.001*
	61-66 Gy	57 (33.3)	13 (15.3)	44 (51.2)	
	≤60 Gy	13 (7.6)	9 (10.6)	4 (4.7)	
Radiologic response (n (%))	Complete response	49 (39.8)	18 (27.3)	31 (54.4)	*0.013*
	Partial response	41 (33.3)	28 (42.4)	13 (22.8)	
	Stable disease	3 (2.4)	1 (1.5)	2 (3.5)	
	Progressive disease	30 (24.4)	19 (28.8)	11 (19.3)	
Mortality at last follow up (n (%))		99 (57.9)	59 (69.4)	40 (46.5)	*0.004*

*Abbreviations: HNSCC* Head and neck squamous cell carcinoma, *RT* Radiotherapy, *n (%)* Number (percentage)

### Baseline and post-RT anemia and hemoglobin levels

The prevalence of anemia (56.5% vs 55.8%) and Hb levels (mean (SD): 12.2 (2.2) g/dL vs 12.5 (2.1) g/dL) before radiotherapy were non-different between the study groups as depicted in Table 3 (*p* > 0.05 for all). Baseline anemia was significantly associated with African American race and higher-grade tumors for the entire study cohort (*p < 0.05* for both) Table S1. Besides, smoking, oral cavity location, locally advanced disease and getting RT in the adjuvant setting were correlated with more anemia in the RT-alone arm (*p < 0.05* for all) Table S2.

After the conclusion of the prescribed RT course, the RT-alone group had significantly lower rates of anemia (44.2% vs. 63.5%, *p* *= 0.017*) with higher mean (SD) Hb level of 12.9 (1.8) g/dL vs 11.98 (2.2) g/dL (*p* = *0.003*), compared to cetuximab plus RT. Both contributed to significantly better improvement of anemia/Hb post RT for RT-alone (*n =* 32, 37.2%) vs. cetuximab plus RT (*n =* 16, 18.8%), *p = 0.007*. When the analysis was restricted to those with pre-existing anemia, 25% had an improvement in the cetuximab plus RT group, which was significantly worse than RT-alone group (58.3%), *p  < 0.001*; with post RT mean (SD) Hb level of 10.9 (1.9) g/dL vs. 12.2 (1.8) g/dL, for both groups respectively, *p = 0.001*. On the other hand, post RT anemia (32.4% vs. 18.4%) and post RT mean (SD) Hb (13.4 (1.7) g/dL vs. 13.8 (1.4) g/dL) were non-different for those without baseline anemia for cetuximab plus RT vs. RT-alone respectively (*p* = 0.26 for both).

**Table 3:** Laboratory investigations, Hb and anemia at baseline and after radiotherapy for HNSCC patients with or without concomitant cetuximab

		All (*n=*171)	Cetuximab plus RT (*n=*85)	RT alone (*n=*86)	*P* value
Hb at baseline (mean (SD))		12.34 (2.17)	12.20 (2.20)	12.48 (2.14)	0.396
Hb after RT (mean (SD))		12.44 (2.04)	11.98 (2.17)	12.90 (1.80)	*0.003*
Anemia at baseline (n (%))		96 (56.1)	48 (56.5)	48 (55.8)	1
Anemia after RT (n (%))		92 (53.8)	54 (63.5)	38 (44.2)	*0.017*
Improvement of anemia or Hb levels (n (%))		48 (28.1)	16 (18.8)	32 (37.2)	*0.007*
CKD (n (%))	No CKD	155 (90.6)	77 (90.6)	78 (90.7)	0.586
	CKD 3	15 (8.8)	8 (9.4)	7 (8.1)	
	ESRD	1 (0.6)	0 (0.0)	1 (1.2)	
Vitamin B12 (n (%))	No data	107 (62.6)	48 (56.5)	59 (68.6)	
	Normal	64 (37.4)	37 (43.5)	27 (31.4)	1
Folate (n (%))	No data	117 (68.4)	52 (61.2)	65 (75.6)	
	Low	2 (1.2)	1 (1.2)	1 (1.2)	1
Iron level (n (%))	Deficiency	9 (5.3)	5 (5.9)	4 (4.7)	0.357
	Overload	17 (9.9)	9 (10.6)	8 (9.3)	
	No data	113 (66.1)	51 (60.0)	62 (72.1)	

*Abbreviations: Hb* Hemoglobin, *HNSCC* Head and neck squamous cell carcinoma, *RT* Radiotherapy, *SD* Standard deviation, *n (%)* Number (percentage), *CKD* Chronic kidney disease, *ESRD* End stage renal disease

### Overall survival with anemia/Hb dynamics

**Fig. 1:**
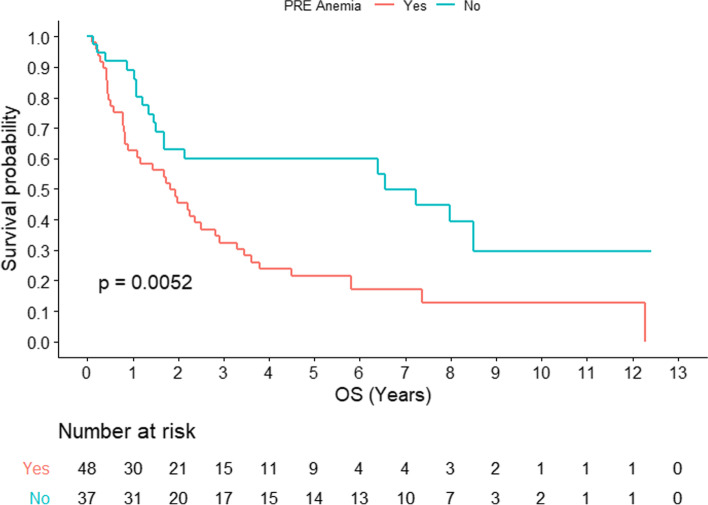
Overall survival for head and neck squamous cell carcinoma with or without baseline anemia for patients receiving concomitant cetuximab (*n =* 85)

**Fig. 2:**
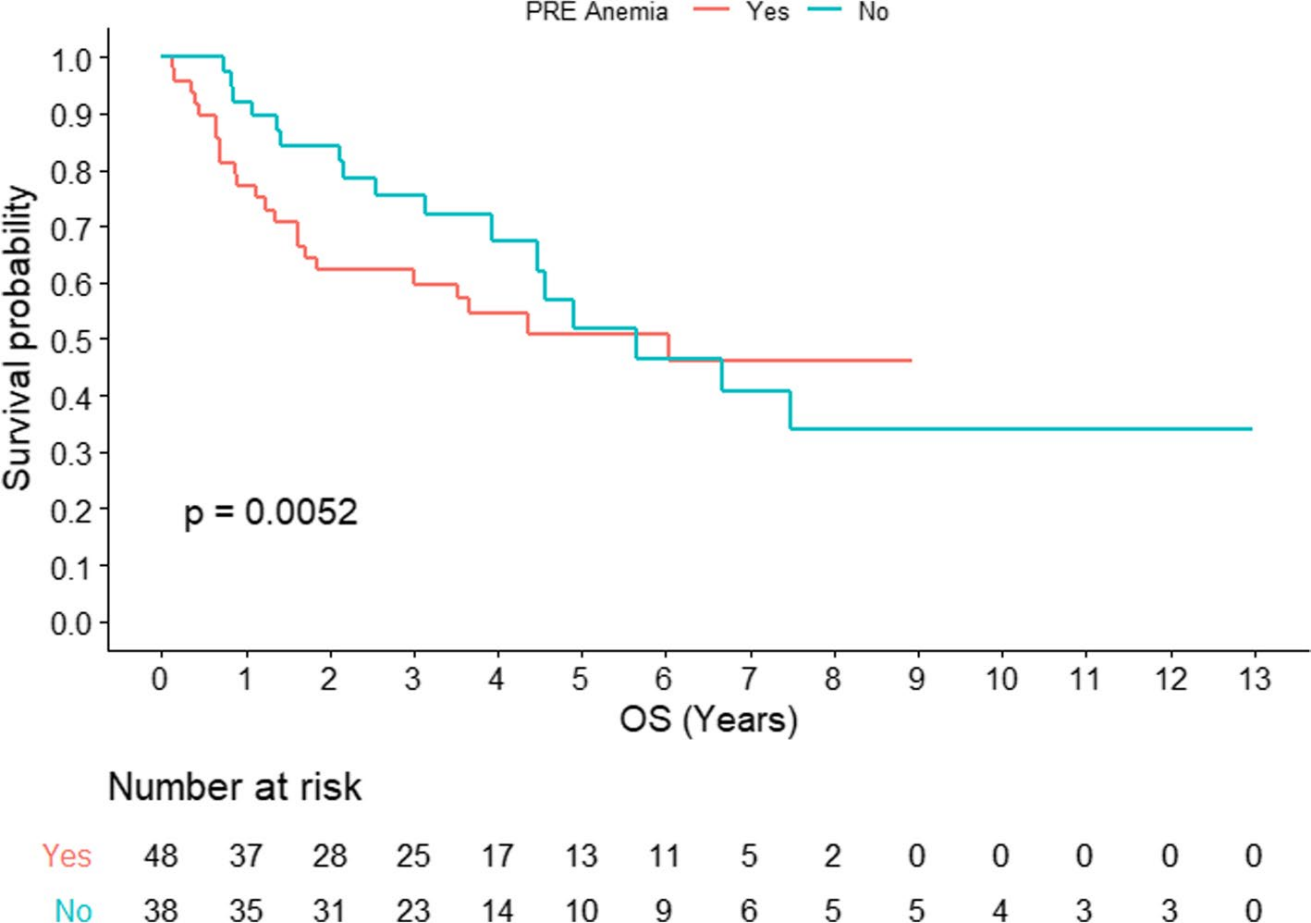
Overall survival for head and neck squamous cell carcinoma with or without baseline anemia for patients receiving radiotherapy alone (*n =* 86)

**Fig. 3:**
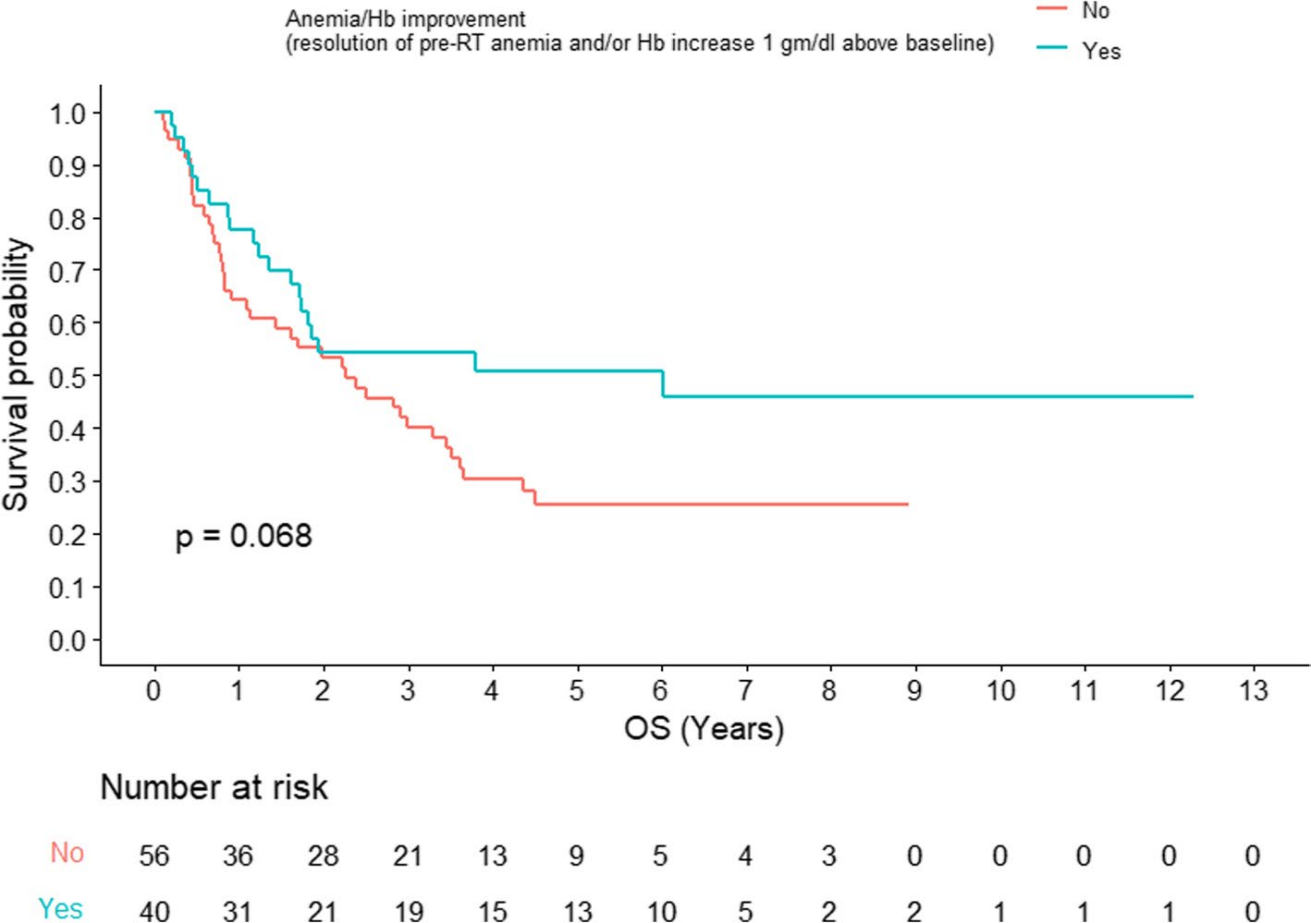
Overall survival for head and neck squamous cell carcinoma with baseline anemia (*n =* 96) with or without post-radiotherapy improvement of anemia/hemoglobin (resolution of pre-RT anemia and/or Hb increase 1 g/dl above baseline)

Baseline anemia was associated with worse OS both in RT-alone (2-year OS: 58% vs 82%; *p = 0.0052*) (Fig. 1) and in RT plus cetuximab (2-year OS: 44% vs 54%; *p = 0.0052*) (Fig. 2). Interestingly, anemia/Hb improvement post-RT for those with baseline anemia failed to reach statistical significance and was marginally associated with improved OS (*p* = 0.068) (Fig. 3).

### Other causes of anemia and sub-group analyses

There was no difference in the rates of G3 CKD (9.4% vs 8.1%) or end-stage renal disease (G5 CKD, 0 vs 1.2%) among both study arms (*p* = 0.59). Vitamin B12 deficiency, folate deficiency and iron disorders were similar between study groups, although this data was only available for limited number of patients as these tests are not routinely ordered for all head and neck cancer patients receiving RT.

In a subgroup analysis, RT-alone was associated with significantly better mean post-RT Hb level when administered both in the adjuvant (12.92 g/dL vs 11.92 g/dL; *p = 0.024*) and in the definitive settings (12.89 g/dL vs 12 g/dL; *p = 0.04*) compared to RT plus cetuximab. Although post-RT anemia level was non-significant in the adjuvant setting (51.3% vs 70.4%; *p* = 0.195) in contrast to definitively RT recipients (38.3% vs 60.3%; *p = 0.04*) for RT-alone vs RT plus cetuximab, overall anemia/Hb improvements were significant only for adjuvant RT (56.4% vs 25.9%; *p = 0.014*), unlike the definitive RT setting (21.3% vs 15.5%; *p* = 0.446) for both study groups respectively.

Of note, the difference in anemia rates after RT among study groups lost significance when stratified by early (57.1% in cetuximab plus RT vs. 41.2% in RT-alone, *p* = 0.33) or locally-advanced stages (65.6% in cetuximab plus RT vs. 48.6% in RT-alone, *p* = 0.15). Nevertheless, locally-advanced demonstrated better post RT mean Hb level (12.85 g/dL vs 11.77 g/dL; *p = 0.01*) and also better anemia improvement (68.6% vs 21.9%; *p  < 0.001*) in RT-alone vs RT plus cetuximab; which was not demonstrated in those of early disease (12.94 vs 12.61 g/dL; *p* = 0.54, and 15.7% vs 9.5%; *p* = 0.71) for RT-alone vs RT plus cetuximab respectively.

### Multivariate analysis for predictors of post-RT anemia

Multivariate analysis showed that cetuximab plus RT was an independent predictor for post-RT anemia (OR 3.16, 95%, CI 1.49–7.05; *p = 0.003*) and low post-RT Hb level (Estimate 0.6, CI 1.13–0.06; *p = 0.029*) (Tables S3–5). Similarly, the use of cetuximab plus RT was deterministic for anemia/Hb improvement after RT conclusion (OR 0.26, CI: 0.10–0.68; *p = 0.007*). The strongest predictor for having anemia at the end of RT was the presence of baseline anemia (OR 7.52, CI 3.44–17.32; *p < 0.001*) adjusting for baseline CCI, alcohol intake, stage, grade, RT setting and dose category. Black race vs white was also independently associated with post-RT anemia (OR 2.81, CI 1.12–7.41; *p = 0.031*). Baseline Hb level was strongly associated with post-RT Hb (Estimate 0.63, CI 0.51–0.74; *p < 0.001*), after accounting for gender and tumor grade. Interestingly, having a locally-advanced tumor was independently prognostic for both post-RT Hb level (Estimate 0.62, CI: 0.05–1.19; *p = 0.034*) as well as for improvement of anemia/Hb (OR 7.19, CI: 2.56–22.45; *p < 0.001*).

## Discussion

Our study shows that patients with nonmetastatic HNSCC that received RT-alone did better than those that received cetuximab with RT in terms of Hb and anemia levels after RT, resulting in higher rates of post-RT anemia/Hb improvement. This outcome was maintained in multivariate analysis after adjusting for other factors. Meanwhile, cetuximab with RT was associated with worse tumor outcomes and survival, albeit stage, tumor site and treatment were not evenly balanced. This contradicts our main hypothesis as we expected that cetuximab would have improved anemia rates in this patient population following RT conclusion. The results are also in disagreement with the study done by Bonner et al., which showed that patients that received cetuximab had lower anemia rates compared to those that received RT-alone. To our knowledge, this is the only study that has done the same comparison, even though patient population, tumor and treatment details were not similar. The study by Bonner et al. was a randomized controlled trial and included a homogenous population with locoregionally advanced HNSCC with similar baseline characteristics among trial arms that received definitive RT. In contrast, our retrospective single institution analysis included all non-metastatic stages and patients received RT in both definitively and adjuvant. We were able to demonstrate rates on anemia/Hb improvement post-RT, which was not depicted by Bonner’s et al. because pre-treatment anemia rates were not reported. According to Bonner et al., the rate of anemia in the cetuximab plus RT group was 3% compared to 13% in RT-alone group (*p < 0.001*). This significant difference persisted after restricting the comparison to G 3–5 of anemia (6% vs. 1%, *p = 0.006*) [14].

A study by Ang et al. compared RT with cisplatin versus RT with cisplatin and cetuximab. The group with cetuximab had a 51% rate of anemia as a complication versus 53% without it, but that was not statistically significant (*p* = 0.55) [18]. In contrast, another study that compared concomitant cetuximab vs carboplatin vs cisplatin revealed significantly lower G3 anemia with cetuximab in HPV-positive oropharyngeal cancer (*p < 0.001*) even though around half of the study population received induction chemotherapy before the RT course [25].

Several other studies compared cetuximab plus RT with chemotherapy plus RT. Magrini et al. reported that patients that received cetuximab with RT had an anemia rate of 6% compared to 50% in those that received RT with cisplatin (*p < 0.001*) in the definitive setting [26]. Hu et al. (2014) reported higher anemia rates overall with the same comparison, but also lower in the group that received cetuximab (48.1% vs. 80.1%, *p < 0.001*) [27]. In contrast, ARTSCAN III: a randomized controlled phase III trial reported non-significant difference between RT with either cisplatin or cetuximab, but their comparison was restricted to G 3–4 anemia [28]. Multiple other studies have reported a similar trend [29–31]. However, the better anemia results for patients that received cetuximab in these studies could be explained by the fact that standard chemotherapy has higher cytotoxic and nephrotoxic effects when compared to cetuximab, rather than a direct effect of cetuximab to promote improvement of anemia.

We proposed that the generally better RT-induced anemia rates that are associated with cetuximab use in Bonner et al., and other studies may have arisen indirectly by lower hepcidin levels contributing to less functional iron deficiency anemia. This effect is thought to be driven at least partially by the lowering of IL-6 levels as a consequence of EGFR inhibition by cetuximab [4, 12, 13]. A study by Wichmann et al. demonstrated significantly lower IL-6 level release, in addition to other pro-inflammatory and pro-angiogenic cytokines by cetuximab on the tissue level, albeit none published on a patient level [32]. Nevertheless, due to the retrospective nature of the study, levels for either IL-6 or hepcidin were not available for the entire study cohort. Of note, we were able to have both baseline iron levels and post-RT levels for only 10 cases (11.7%) in the cetuximab with RT and 4 cases (4.6%) that received RT-alone. Interestingly, the change of iron level parameters (iron, ferritin and total iron binding capacity) following RT does not seem to be consistently influenced by cetuximab use and was not associated with post RT anemia or Hb levels as demonstrated in Tables S6–7. Although numbers prevented a formal comparison, this goes in line with the primary outcome of this study that cetuximab did not significantly lower RT-induced anemia compared to RT-alone for the investigated cohort. We strongly recommend recording baseline and post-RT iron studies and anemia levels as well as hepcidin and IL-6 levels whenever possible for all prospective studies utilizing cetuximab concomitant with RT so that we can have a definite conclusion.

The better toxicity profile supported by the efficacy results of the Bonner et al. study encouraged the administration of cetuximab with RT as a treatment arm in de-escalation trials for the HPV-positive oropharyngeal cancer. Gillison et al. reported 0% G3–4 acute anemia compared to 2.8% for cetuximab vs. cisplatin (*p = 0.0009*) [33]. Similar results were portrayed in the De-ESCALate randomized trial with 0% vs. 2% for G3–5 anemia [34]. The lack of any G3 or above acute anemia in these recent major trials (0%) reinforces indirectly how cetuximab may protect against, or at least is not associated with an increase in, RT-induced anemia.

Of note, baseline anemia was associated with significantly worse overall survival, which was consistent for both study arms. This is in agreement with previous studies addressing both definitive [7] and adjuvant [8] radiotherapy settings. On the other hand, improvement of anemia/Hb post-RT was not translated into better survival. This underscores the importance of studying head and neck squamous cell carcinoma tumors for patient with baseline anemia on the molecular level in the era of precision medicine. The independent prognostic effect of locally advanced stage on the improvement of anemia/Hb deserves further dedicated studies.

The results of our study must be interpreted with caution. Given the retrospective nature of our research and the relatively limited number of patients, some characteristics were not well distributed between the two groups. Even though anemia rates prior to treatment were similar, the fact that the group receiving cetuximab had more patients with locally advanced tumors may have contributed to a poorer outcome overall in this group, including anemia rates after treatment, albeit our findings support the opposite. It should also be noted that our study included patients treated both in adjuvant and definitive settings to increase data availability, which could add confounding variables related to prior surgical intervention. RTOG-0234 is the only randomized trial that administered cetuximab in the postoperative RT setting for high-risk HNSCC cases. However, cetuximab was used in both arms of the study combined with cisplatin or docetaxel (G2–4 anemia 15% vs. 6%, respectively) [15]. The RT-alone group had a non-significant trend towards more patients treated in an adjuvant setting and it is possible that the better improvement of anemia/Hb after RT was influenced by recovery from perioperative anemia. Furthermore, patients in the cetuximab plus RT group had a higher proportion of poorly differentiated tumors and have received higher doses of RT, which may also have contributed to worse anemia rates rather than a real harmful effect of cetuximab compared to RT-alone as it may be assumed in our results. Lastly, our study was limited by the lack of availability of many laboratory results for over 60% of study subjects within the predetermined timeframe as these tests are not routinely ordered for all patients. This was particularly troublesome when trying to compare causes of anemia by measuring vitamin B12, folate, and iron studies which would have enhanced the robustness of our findings taking in consideration that no previous studies discussed this until now.

## Conclusions

Cetuximab did not prevent or improve anemia related to RT in our study, which is not consistent with the study by Bonner et al. [9]. The potential explanations for these findings are discussed above but may be attributed to the heterogeneity of our study population, staging and treatment imbalances; in addition, to the retrospective nature of data gathering. Our findings are not definitive and further studies are needed to better elucidate the role of cetuximab in the prevention of anemia during RT if any.

## Supplementary Information

**Additional file 1.**

## Data Availability

The datasets generated and/or analyzed during the current study are not publicly available due IRB restrictions but are available from the corresponding author upon reasonable request.

## Abbreviations

RT: Radiation therapy
Hb: Hemoglobin
EGFR: Epidermal growth factor receptor
HER-1: Human epidermal growth factor-1
TGF-⍺: Transforming growth factor alpha
IL-6: Interleukin 6
HNSCC: Head and neck squamous cell carcinoma
CCI: Charlson comorbidity index
HPV: Human papilloma virus
CKD: Chronic kidney disease
G: Grade
OS: Overall survival
SD: Standard deviation
IQR: Interquartile range
OR: Odds ratio
CI: Confidence intervals

## References

[1] Rodgers GM, Becker PS, Blinder M, Cella D, Chanan-Khan A, Cleeland C (2012). Cancer- and chemotherapy-induced Anemia. J Natl Compr Cancer Netw.

[2] Harrison LB, Shasha D, White C, Ramdeen B (2000). Radiotherapy-associated Anemia: the scope of the problem. Oncologist.

[3] Harrison L, Shasha D, Shiaova L, White C, Ramdeen B, Portenoy R (2001). Prevalence of Anemia in Cancer Pateints undergoing radiation therapy. Semin Oncol.

[4] Harrison L, Shasha D, Homel P (2002). Prevalence of anemia in cancer patients undergoing radiotherapy: prognostic significance and treatment. Oncology..

[5] Caro JJ, Salas M, Ward A, Goss G (2001). Anemia as an independent prognostic factor for survival in patients with cancer: a systemic, quantitative review. Cancer..

[6] Kumar P (2000). Impact of Anemia in patients with head and neck Cancer. Oncologist..

[7] Warde P, O’Sullivan B, Bristow RG (1998). T1/T2 glottic cancer managed by external beam radiotherapy: the influence of pretreatment hemoglobin on local control. Int J Radiat Oncol Biol Phys.

[8] Rades D, Seidl D, Janssen S, Wollenberg B, Hakim SG, Schild SE (2016). The effect of low hemoglobin levels on outcomes of radiotherapy following microscopically complete resection of locally advanced SCCHN: implications for the future. J Craniomaxillofac Surg.

[9] Gilreath JA, Stenehjem DD, Rodgers GM (2014). Diagnosis and treatment of cancer-related anemia. Am J Hematol.

[10] Overgaard J, Hoff CM, Hansen HS (2018). DAHANCA 10 – effect of darbepoetin alfa and radiotherapy in the treatment of squamous cell carcinoma of the head and neck. A multicenter, open-label, randomized, phase 3 trial by the Danish head and neck cancer group. Int J Radiat Oncol Biol Phys.

[11] Zimmermann M, Zouhair A, Azria D (2006). The epidermal growth factor receptor (EGFR) in head and neck cancer: its role and treatment implications. Radiat Oncol.

[12] Ray K, Ujvari B, Ramana V, Donald J (2018). Cross-talk between EGFR and IL-6 drives oncogenic signaling and offers therapeutic opportunities in cancer. Cytokine Growth Factor Rev.

[13] Grellier N, Deray G, Yousfi A, Khodari W, Bouaita R (2015). Belkacemi Y. Carence martiale fonctionnelle, inflammation et fatigue après radiothérapie Functional iron deficiency, inflammation and fatigue after radiotherapy. Bull Cancer.

[14] Bonner JA, Harari PM, Giralt J, Azarnia N, Shin DM, Cohen RB, Jones CU, Sur R, Raben D, Jassem J, Ove R, Kies MS, Baselga J, Youssoufian H, Amellal N, Rowinsky EK, Ang KK (2006). Radiotherapy plus cetuximab for squamous-cell carcinoma of the head and neck. N Engl J Med.

[15] Harari PM, Harris J, Kies MS, Myers JN, Jordan RC, Gillison ML, Foote RL, Machtay M, Rotman M, Khuntia D (2014). Postoperative Chemoradiotherapy and Cetuximab for high-risk squamous cell carcinoma of the head and neck: radiation therapy oncology group RTOG-0234. J Clin Oncol.

[16] NCCN Clinical Practice Guidelines in Oncology: Head and Neck Cancers, Version 3; 2021. Available at: https://www.nccn.org/professionals/physician_gls/pdf/head-and-neck.pdf. Accessed June 21, 2021.

[17] Petrelli F, Coinu A, Riboldi V, Borgonovo K, Ghilardi M, Cabiddu M, Lonati V, Sarti E, Barni S (2014). Concomitant platinum-based chemotherapy or cetuximab with radiotherapy for locally advanced head and neck cancer: a systematic review and meta-analysis of published studies. Oral Oncol.

[18] Ang KK, Zhang Q, Rosenthal DI, Nguyen-Tan PF, Sherman EJ, Weber RS (2014). Randomized phase III trial of concurrent accelerated radiation plus cisplatin with or without cetuximab for stage III to IV head and neck carcinoma: RTOG 0522. J Clin Oncol.

[19] Vermorken JB, Mesia R, Rivera F, Remenar E, Kawecki A, Rottey S (2008). Platinum-based chemotherapy plus cetuximab in head and neck cancer. N Engl J Med.

[20] Amin MB, Edge SB, Greene FL (2017). AJCC Cancer staging manual.

[21] El-Naggar AK, Westra WH (2012). p16 expression as a surrogate marker for HPV-related oro-pharyngeal carcinoma: a guide for interpretative relevance and consistency. Head Neck.

[22] Schwartz, L.H.; Litière, S.; De Vries, E.; Ford, R.; Gwyther, S.; Mandrekar, S.; Shankar, L.; Bogaerts, J.; Chen, A.; Dancey, J.; et al. RECIST 1.1—update and clarification: from the RECIST committee. Eur J Dermatol Cancer 2016, 62, 132–137. 10.1016/j.ejca.2016.03.081 PMC5737828 27189322

[23] KDIGO. Summary of recommendation statements. Kidney Int. 2013;3 (Suppl):5. 10.1038/kisup.2012.77 PMC4284512 25598998

[24] Anemia: WHO, UNICEF, UNU (2001). Iron deficiency anaemia: assessment, prevention, and control. A guide for programme managers.

[25] Nien HH, Sturgis EM, Kies MS, El-Naggar AK, Morrison WH, Beadle BM (2016). Comparison of systemic therapies used concurrently with radiation for the treatment of human papillomavirus- associated oropharyngeal cancer. Head Neck.

[26] Magrini SM, Buglione M, Corvo R, Pirtoli L, Paiar F, Ponticelli P (2016). Cetuximab and radiotherapy versus cisplatin and radiotherapy for locally advanced head and neck Cancer: a randomized phase II trial. J Clin Oncol.

[27] Hu MH, Wang LW, Lu HJ, Chu PY, Tai SK, Lee TL (2014). Cisplatin-based chemotherapy versus cetuximab in concurrent chemoradiotherapy for locally advanced head and neck cancer treatment. Biomed Res Int.

[28] Gebre-Medhin M, Brun E, Engstrom P (2021). ARTSCAN III: a randomized phase III study comparing chemoradiotherapy with cisplatin versus cetuximab in patients with locoregionally advanced head and neck squamous cell cancer. J Clin Oncol.

[29] Levy A, Blanchard P, Bellefqih S, Brahimi N, Guigay J, Janot F (2014). Concurrent use of cisplatin or cetuximab with definitive radiotherapy for locally advanced head and neck squamous cell carcinomas. Strahlenther Onkol.

[30] Lefebvre JL, Pointreau Y, Rolland F, Alfonsi M, Baudoux A, Sire C (2013). Induction chemotherapy followed by either chemoradiotherapy or bioradiotherapy for larynx preservation: the TREMPLIN randomized phase II study. J Clin Oncol.

[31] Dornoff N, Weiss C, Rodel F, Wagenblast J, Ghanaati S, Atefeh N (2015). Re-irradiation with cetuximab or cisplatin-based chemotherapy for recurrent squamous cell carcinoma of the head and neck. Strahlenther Onkol.

[32] Wichmann G, Cedra S, Schlegel D, Kolb M, Wiegand S, Boehm A (2017). Cilengitide and Cetuximab reduce cytokine production and Colony formation of head and neck squamous cell carcinoma cells ex vivo. Anticancer Res.

[33] Gillison ML, Trotti AM, Harris J, Eisbruch A, Harari PM, Adelstein DJ (2019). Radiotherapy plus cetuximab or cisplatin in human papillomavirus-positive oropharyngeal cancer (NRG oncology RTOG 1016): a randomised, multicentre, non-inferiority trial. Lancet..

[34] Mehanna H, Robinson M, Hartley A, Kong A, Foran B, Fulton-Lieuw T (2019). Radiotherapy plus cisplatin or cetuximab in low-risk human papillomavirus-positive oropharyngeal cancer (De- ESCALaTE HPV): an open-label randomised controlled phase 3 trial. Lancet..

