# Supplementary material for: Cetuximab and anemia prevention in head and neck cancer patients undergoing radiotherapy
Source: BMC Cancer. 2022 Jun 7;22:626. doi: 10.1186/s12885-022-09708-9 (PMC9175328; doi:10.1186/s12885-022-09708-9)
Supplement: Supplementary file 1 — Additional file 1. [file 12885_2022_9708_MOESM1_ESM.docx]

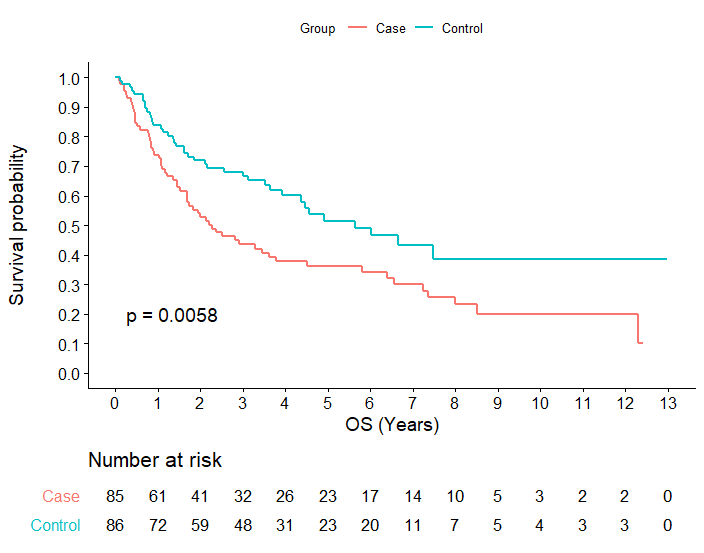


Figure: S1: Overall survival for head and neck squamous cell carcinoma for patients receiving concomitant cetuximab (cases, n=85) vs RT-alone (control, n=86)


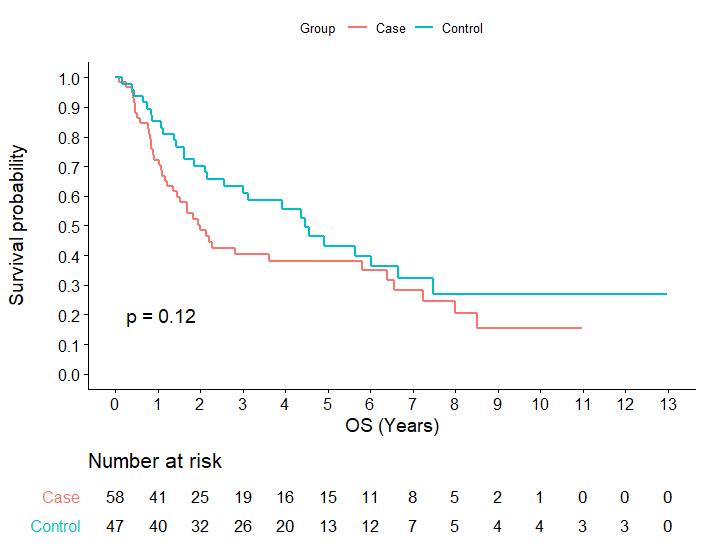


Figure: S2: Overall survival for head and neck squamous cell carcinoma receiving RT in the definitive setting alone (control, n=47) or with concomitant cetuximab (cases, n=58)

Table S1. Baseline demographic and tumor characteristics associated with baseline anemia for HNSCC patients receiving radiotherapy with cetuximab

|  |  | All (n=85) | Anemia at baseline (n=48) | No anemia at baseline (n=37) | P value |
| --- | --- | --- | --- | --- | --- |
| Mean age at Diagnosis in years [SD] |  | 65.71 (12.20) | 65.94 (11.66) | 65.41 (13.03) | 0.843 |
| Age group at Diagnosis in years (n (%)) | <50 | 8 (9.4) | 4 (8.3) | 4 (10.8) | 0.639 |
|  | 50-70 | 44 (51.8) | 27 (56.2) | 17 (45.9) |  |
|  | >70 | 33 (38.8) | 17 (35.4) | 16 (43.2) |  |
| Gender (n (%)) | Female | 18 (21.2) | 12 (21.2) | 6 (16.2) | 0.475 |
|  | Male | 67 (78.8) | 36 (78.8) | 31 (83.8) |  |
| Race (n (%)) | Black | 23 (27.1) | 20 (41.7) | 3 (8.1) | 0.001 |
|  | White | 60 (70.6) | 26 (54.2) | 34 (91.9) |  |
|  | Other | 2 (2.4) | 2 (4.2) | 0 (0) |  |
| Smoking (n (%)) | Never | 15 (17.6) | 7 (14.6) | 8 (21.6) | 0.514 |
|  | Former | 42 (49.4) | 23 (47.9) | 19 (51.4) |  |
|  | Active | 28 (32.9) | 18 (37.5) | 10 (27.0) |  |
| Alcohol use (n (%)) | Never | 28 (32.9) | 14 (29.2) | 14 (37.8) | 0.076 |
|  | Occasional | 25 (29.4) | 11 (22.9) | 14 (37.8) |  |
|  | Frequent | 32 (37.6) | 23 (47.9) | 9 (24.3) |  |
| Tumor site (n (%)) | Oral cavity | 11 (12.9) | 8 (16.7) | 3 (8.1) | 0.079 |
|  | Oropharynx | 56 (65.9) | 27 (56.2) | 29 (78.4) |  |
|  | Hypopharynx | 1 (1.2) | 0 (0) | 1 (2.7) |  |
|  | Larynx | 17 (20.0) | 13 (27.1) | 4 (10.8) |  |
| Tumor staging (n (%)) | Early | 21 (24.7) | 8 (16.7) | 13 (35.1) | 0.088 |
|  | Locally advanced | 64 (75.3) | 40 (83.3) | 24 (64.9) |  |
| Tumor grade of differentiation (n (%)) | Well | 22 (45.8) | 11 (36.7) | 11 (61.1) | 0.047 |
|  | Moderate | 23 (47.9) | 18 (60.0) | 5 (27.8) |  |
|  | Poor | 3 (6.25) | 1 (3.3) | 2 (11.1) |  |
| HPV status (oropharyngeal cancers) (n (%)) | Positive | 21 (60.0) | 6 (40.0) | 15 (75.0) | 0.054 |
|  | Negative | 14 (40.0) | 9 (60.0) | 5 (25.0) |  |

Abbreviations: HNSCC, head and neck squamous cell carcinoma; RT, radiotherapy; SD, standard deviation; n (%), number (percentage); HPV, human papilloma virus

Table S2. Baseline demographic and tumor characteristics associated with baseline anemia for HNSCC patients receiving radiotherapy alone

|  |  | All (n=86) | Anemia at baseline (n=48) | No anemia at baseline (n=38) | P value |
| --- | --- | --- | --- | --- | --- |
| Mean age at Diagnosis in years [SD] |  | 65.31 (10.29) | 67.02 (10.2) | 63.16 (10.12) | 0.084 |
| Age group at Diagnosis in years (n (%)) | <50 | 3 (3.5) | 1 (2.1) | 2 (5.3) | 0.404 |
|  | 50-70 | 57 (66.3) | 30 (62.5) | 27 (71.1) |  |
|  | >70 | 26 (30.2) | 17 (35.4) | 9 (23.7) |  |
| Gender (n (%)) | Female | 19 (22.2) | 12 (25.0) | 7 (18.4) | 0.639 |
|  | Male | 67 (77.9) | 36 (75.0) | 31 (81.6) |  |
| Race (n (%)) | Black | 27 (31.4) | 20 (41.7) | 7 (18.4) | 0.022 |
|  | White | 57 (66.3) | 26 (54.2) | 31 (81.6) |  |
|  | Other | 2 (2.3) | 2 (4.2) | 0 (0) |  |
| Smoking (n (%)) | Never | 15 (17.4) | 4 (8.3) | 11 (28.9) | 0.085 |
|  | Former | 45 (52.3) | 32 (66.7) | 13 (34.2) |  |
|  | Active | 26 (30.2) | 12 (25.0) | 14 (36.8) |  |
| Alcohol use (n (%)) | Never | 29 (33.7) | 16 (33.3) | 13 (34.2) | 0.085 |
|  | Occasional | 27 (31.4) | 11 (22.9) | 16 (42.1) |  |
|  | Frequent | 30 (34.9) | 21 (43.8) | 9 (23.7) |  |
| Tumor site (n (%)) | Oral cavity | 18 (20.9) | 15 (31.2) | 3 (7.9) | 0.047 |
|  | Oropharynx | 26 (30.2) | 13 (27.1) | 13 (34.2) |  |
|  | Hypopharynx | 1 (1.2) | 0 (0) | 1 (2.6) |  |
|  | Larynx | 41 (47.7) | 20 (41.7) | 21 (55.3) |  |
| Tumor staging (n (%)) | Early | 51 (59.3) | 19 (39.6) | 32 (84.2) | <0.001 |
|  | Locally advanced | 35 (40.7) | 29 (60.4) | 6 (15.8) |  |
| Tumor grade of differentiation (n (%)) | Well | 5 (9.8) | 4 (11.8) | 1 (5.9) | 0.042 |
|  | Moderate | 39 (76.5) | 28 (82.3) | 11 (64.7) |  |
|  | Poor | 7 (13.7) | 2 (5.9) | 5 (29.4) |  |
| HPV status (oropharyngeal cancers) (n (%)) | Positive | 16 (76.2) | 6 (54.5) | 10 (100.0) | 0.054 |
|  | Negative | 5 (23.8) | 5 (45.5) | 0 (00.0) |  |

Abbreviations: HNSCC, head and neck squamous cell carcinoma; RT, radiotherapy; SD, standard deviation; n (%), number (percentage); HPV, human papilloma virus

Table S3. Multivariate analysis Cox regression models results for predictors of Hb level after RT for the entire study cohort (n=171)

|  |  | **Hb after RT^i^** | | |
| --- | --- | --- | --- | --- |
|  | *Predictors* | *Estimates* | *CI* | P value |
|  | Study group [cetuximab plus RT vs RT-alone] | -0.60 | -1.13 – -0.06 | 0.029 |
|  | Hb at baseline | 0.63 | 0.51 – 0.74 | <0.001 |
|  | Tumor staging [Locally advanced vs Early] | 0.62 | 0.05 – 1.19 | 0.034 |
|  | RT dose category [70-72 Gy vs 61-66 Gy] | 0.63 | 0.09 – 1.16 | 0.022 |

^I^ Adjusted for gender and tumor grade of differentiation

Abbreviations: Hb, hemoglobin; RT, radiotherapy

Table S4. Multivariate analysis Cox regression models results for predictors of anemia level after RT for the entire study cohort (n=171)

|  | **Anemia after RT^i^** | | |
| --- | --- | --- | --- |
| *Predictors* | *Odds Ratios* | *CI* | P value |
| Study group [cetuximab plus RT vs RT-alone] | 3.16 | 1.49 – 7.05 | 0.003 |
| Anemia at baseline [yes vs no] | 7.52 | 3.44 – 17.32 | <0.001 |
| Race [Black vs White] | 2.81 | 1.12 – 7.41 | 0.031 |

^I^ Adjusted for alcohol use

Abbreviations: RT, radiotherapy

Table S5. Multivariate analysis Cox regression models results for predictors of improvement of anemia/Hb after RT for the entire study cohort (n=171)

|  | **Improvement of anemia/Hb after RT^i^** | | |
| --- | --- | --- | --- |
| *Predictors* | *Odds Ratios* | *CI* | P value |
| Study group [cetuximab plus RT vs RT-alone] | 0.26 | 0.10 – 0.68 | 0.007 |
| Anemia at baseline [yes vs no] | 7.39 | 2.56 – 25.27 | 0.001 |
| Tumor grade of differentiation [Poor vs Well] | 20.85 | 3.05 – 169.68 | 0.003 |
| Tumor staging [Locally advanced vs Early] | 7.19 | 2.56 – 22.45 | <0.001 |

^I^ Adjusted for alcohol RT dose category

Abbreviations: Hb, hemoglobin; RT, radiotherapy

Table S6. Hemoglobin and anemia in relation to iron level dynamics for cases with available iron levels at baseline and after radiotherapy for cases receiving radiotherapy plus cetuximab (n=10)

| Hgb at baseline | Anemia at baseline | Hgb after RT | Anemia after RT | MCV after RT | Ferritin at baseline | Ferritin after RT | Iron at baseline | Iron after RT | TIBC at baseline | TIBC after RT |
| --- | --- | --- | --- | --- | --- | --- | --- | --- | --- | --- |
| 8.2 | Yes | 10 | Yes | 79.4 | 59 | 93 | <10 | <10 | 238 | 183 |
| 12 | Yes | 12.3 | Yes | 94.6 | 734 | 286 | 34 | 17 | 143 | 186 |
| 11.4 | Yes | 13.7 | No | 88.5 | 941 | 188 | 38 | 83 | 356 | 317 |
| 10.6 | Yes | 10.1 | Yes | 80.5 | 200 | 77 | 29 | 30 | 444 | 386 |
| 9.7 | Yes | 8.4 | Yes | 78.2 | 122 | 25 | 53 | 19 | 432 | 462 |
| 9.9 | Yes | 10.3 | Yes | 85.8 | N/A | 381 | 22 | 76 | 295 | 353 |
| 9.3 | Yes | 12.4 | Yes | 91.5 | 319 | 360 | 37 | 98 | 287 | 401 |
| 14 | Yes | 13.5 | No | 88.7 | 292 | 310 | 67 | 46 | 364 | 373 |
| 11.8 | Yes | 10.9 | Yes | 84.3 | 413 | N/A | 63 | 26 | 195 | 241 |
| 12.7 | Yes | 11 | Yes | 96 | N/A | 644 | 127 | 69 | 216 | 279 |

Abbreviations: Hb, hemoglobin; RT, radiotherapy; MCV, mean corpuscular volume; N/A, not available; TIBC, total iron binding capacity

Table S7. Hemoglobin and anemia in relation to iron level dynamics for cases with available iron levels at baseline and after radiotherapy for cases receiving radiotherapy alone (n=4)

| Hb at baseline | Anemia at baseline | Hb after RT | Anemia after RT | MCV after RT | Ferritin at baseline | Ferritin after RT | Iron at baseline | Iron after RT | TIBC at baseline | TIBC after RT |
| --- | --- | --- | --- | --- | --- | --- | --- | --- | --- | --- |
| 11.9 | Yes | 10.7 | Yes | 91.7 |  | 869 | 91 | 70 | 353 | 279 |
| 9.3 | Yes | 7.7 | Yes | 93.6 | 264 | 1479 | 39 | 86 | 164 | 169 |
| 9.8 | Yes | 11.7 | Yes | 89 | 134 | 69 | 67 | 26 | 351 | 290 |
| 11.2 | Yes | 12.7 | Yes | 87.1 | N/A | N/A | 43 | 77 | 333 | 370 |

Abbreviations: Hb, hemoglobin; RT, radiotherapy; MCV, mean corpuscular volume; N/A, not available; TIBC, total iron binding capacity
